# Laser Doppler Spectrum Analysis Based on Calculation of Cumulative Sums Detects Changes in Skin Capillary Blood Flow in Type 2 Diabetes Mellitus

**DOI:** 10.3390/diagnostics11020267

**Published:** 2021-02-09

**Authors:** Igor Kozlov, Evgeny Zherebtsov, Galina Masalygina, Konstantin Podmasteryev, Andrey Dunaev

**Affiliations:** 1 Research and Development Center of Biomedical Photonics, Orel State University, 302026 Orel, Russia; evgenii.zherebtsov@oulu.fi (E.Z.); asms-orel@mail.ru (K.P.); inohvat@yandex.ru (A.D.); 2 Optoelectronics and Measurement Techniques Unit, University of Oulu, 90570 Oulu, Finland; 3 Orel Regional Clinical Hospital, 302028 Orel, Russia; kovalina68@ya.ru

**Keywords:** laser Doppler flowmetry, non-invasive optical diagnostics, cumulative sum, power spectrum, heating test, diabetes mellitus type 2

## Abstract

In this article, we introduce a new method of signal processing and data analysis for the digital laser Doppler flowmetry. Our approach is based on the calculation of cumulative sums over the registered Doppler power spectra. The introduced new parameter represents an integral estimation for the redistribution of moving red blood cells over the range of speed. The prototype of the device implementing the technique is developed and tested in preliminary clinical trials. The methodology was verified with the involvement of two age groups of healthy volunteers and in a group of patients with type 2 diabetes mellitus. The main practical result of the study is the development of a set of binary linear classifiers that allow the method to identify typical patterns of the microcirculation for the healthy volunteers and diabetic patients based on the presented diagnostic algorithm.

## 1. Introduction

The blood microcirculation (BM) performs a crucial role for the life support of every type of living tissue in the human body. The BM supports gases and nutrients exchange, delivering of immune cells, and temperature regulation. The BM makes a major contribution to the overall flow resistance of the blood vessels, being the main actor in the regulation of the blood pressure. The functional state of human tissues directly depends on the state of elementary BM units (capillaries, plexus, minor arterioles, and venules). It is well-known [1] that the microcirculatory bed is composed of blood vessels of the certain types with a particular specialisation: arterioles, capillaries, venules and arterio-venular anastomoses (AVA). AVA, together with the smooth muscle structures acts as a physiological valve that redirects the micro blood flow either through the capillary plexus or bypassing one. The process orchestrated by the physiological regulation from the endothelium, neural activity, myogenic contraction and relaxation, as well as by the modulation from the breathing and heart activity creates characteristic patterns of the blood perfusion fluctuations [2,3].

The disruptions in BM significantly contribute to the development of many diseases and syndromes. One of the most common and widespread diseases with a plethora of severe complications affecting BM is diabetes mellitus type 2 (T2DM). According to forecasts, the number of patients with T2DM is projected to constantly grow in the near future to achieve 500 million people by 2030 [4,5]. Common complications of T2DM are ulcers and lesions on the feet and toes, as well as the development of necrosis due to the trophic disorders of the affected tissues. Optical, non-invasive diagnostics offer a good range of methods for the evaluation of the functional insufficiency in the components of tissue vitality including blood microcirculation system. The methods are based on such approaches as fluorescence spectroscopy [6], diffuse reflectance spectrocopy [7,8,9], speckle contrast imaging [10], photopletismography [11], videocapillaroscopy [12] and methods based on coherent light scattering and optical coherence tomography [13,14].

One well-established method for the non-invasive measurements of the blood perfusion in vivo is the laser Doppler flowmetry (LDF). The theoretical basis for the technique is based on the statistical analysis of the laser radiation scattered in the scattering medium with the moving light-scattering elements. For the case of the measurements in the living tissue, the static scattering structures are the cells and static tissue layers, whereas the moving particles are the locomotive blood cells in the flowing capillaries and blood microvessels. The subtle frequency shift caused by an optical Doppler effect is usually detected by the couple of photodiodes operating in the photomixing regime [15,16]. This method has a sufficient time resolution and depth of probing (about 1 mm [17]) to register the blood microflow fluctuations of different origin, as well as the average level of blood perfusion. The range of applications of the non-invasive diagnostics by the LDF method covers the topics of relationship between oxygen saturation and blood flow [18], dental pulp blood flow [19], blood flow analysis in cases of type 1 diabetes mellitus [20] and rheumatic diseases [21], combining LDF and endoscopy during surgery interventions [22,23], nonlinear blood flow dynamics analysis in a various application [24,25], analysis of transdermal transport of drugs [26] and registration of arterial hypertension associated changes in microcirculation [27]. Numerically, the resulting value for the blood perfusion in the LDF technique is commonly given by the expression:

(1) $$PU=\int_{f_{min}}^{f_{max}}f\cdot S\left(f\right)df,$$

where S(f) is the power spectrum of the photocurrent given as a function of the frequency, *f*; $f_{min}$, $f_{max}$ are the limits of integration over the region of the power spectrum corresponding to the physiological range of the RBC speed in the blood capillaries. In several recent studies, more advanced techniques on the LDF signal processing were presented [28,29]. The power spectrum analysis within selected ranges of integration allowed Fredriksson et al., to estimate the three ranges of RBC speed in the diagnostic volume of a fibre optical probe. Taking into account the RBC speed distribution and light scattering phase function, a similar approach has been applied for the laser Doppler spectrum decomposition [30,31].

From the very early studies on the LDF technique development, it has been revealed that the distribution of the power spectrum amplitudes over the dimension of frequency changes to a considerable degree, and represents the frequency distribution of intensities for the Doppler shifted optical components scattered on moving RBC [32,33]. In many clinical studies, it has been demonstrated that the information is of substantial diagnostic value for the quantitative characterisation of the microcirculation disorders [34,35,36,37,38]. As an example, the LDF signal recorded with the power spectrum integration up to the frequency of 3000 Hz demonstrated a better signal-to-noise ratio and presented overall quality of diagnostics of the dental pulp vitality than using the full range of the bandwidth [39]. The power spectrum properties for the purposes of the verification of the LDF technique were also studied in liquid phantoms where the interrelation between the distribution of the speed of scattering particles with the registered power spectrum of photocurrent from the detector was also confirmed [40]. Also, the effects of local pressure on the optical probe to the skin surface [34] and breath-holding test [41] on the broadening of the Doppler spectra were studied. Despite the recent interest several research groups on the diagnostic potential of LDF measurements with a more sophisticated analysis of the detector power spectrum, the topic has still not been well understood and dealt with in depth.

In that respect, the aim of this study was to elaborate the concept of a new feature space extracted from the Doppler power spectrum that will increase the diagnostic capability of the LDF method for analysis of blood microcirculation disorders in patients with T2DM.

## 2. Materials and Methods

**Figure 1:**
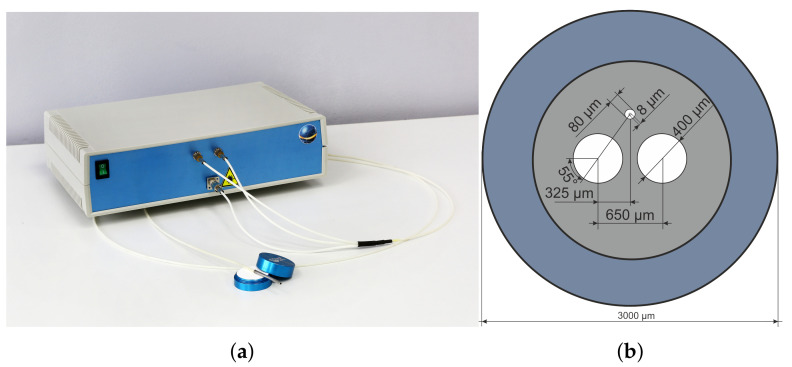
In-house built prototype of laser Doppler flowmeter with 2 mW of output light power, a signal amplifying, filtration unit, 50 kHz sample rate per channel (**a**) and fibre geometrical configuration (**b**).

**Figure 2:**
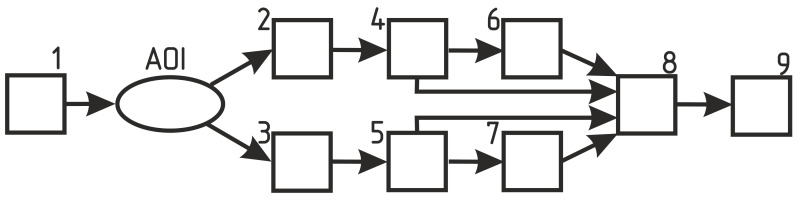
The schematic diagram of the developed laser Doppler flowmetry measuring setup. 1— infrared laser diode; 2, 3—light-to-current conversion and amplification modules; 4, 5—low-frequency filters; 6, 7—high-frequency filters; 8—data acquisition board; 9—PC with LabVIEW-based application.

To implement the LDF signal registration, an in-house built setup was designed and tested (Figure 1). The block diagram of the device is shown in Figure 2. Infra-red laser diode (LPS-785-FC, Thorlabs, USA) (1) with a central radiation wavelength of 785 nm was used for the local illumination of the area of interest (AOI) with 2 mW of output light power. The channel of the electronic signal processing consisted of light-to-current converting and amplifying modules (2, 3), low-frequency (4, 5) and high-frequency (6, 7) filters and a unit for the stabilised power supply.

The analog-to-digital conversion was implemented by a USB 6211 (National Instruments™,Austin, Texas, USA) data acquisition board (8) with a sampling rate of 50 kHz per channel. LabVIEW™-based PC application (9) was developed for the device control, data acquisition and pre-processing. The period of cycle with the recording of time series containing 2500 data points of photocurrent and the power spectrum calculation was set to be 0.05 s, with the corresponding sampling rate of 20 Hz for the output calculated perfusion. The cut-off frequency for the calculation of the power spectrum was set to 12,800 Hz with the possible increase of this parameter up to the Nyquist frequency limit. 

For the blood perfusion calculations over the sub-regions of the power spectrum, the following expression has been used:

(2) $$PU=\frac{K}{i_{dc}^{2}}\int_{f_{min}}^{f_{max}}f\cdot S\left(i_{ac2}\left(t\right)-i_{ac1}\left(t\right)\right)df,$$

where *K*—the static coefficient of gain; $i_{dc}\left(t\right)$—the d.c. component of the photocurrent; $i_{ac1}\left(t\right)$, $i_{ac2}\left(t\right)$—the a.c. components of the photocurrents in the two input photo-detecting sub-channels (the implementation of the differential measuring technique with the subtraction of $i_{ac1}\left(t\right)$ and $i_{ac2}\left(t\right)$ significantly reduces the motion artefacts from the fibre optical probe); *S*—the procedure of the power spectrum calculation from the accumulated array of the subtracted values of $i_{ac1}\left(t\right)$ and $i_{ac2}\left(t\right)$. The key functionality of the developed software was the ability to save on disk the calculated power spectra for every sample for further post-processing.

In addition to conventional perfusion units, an estimation of the blood cell speed 〈v〉 [42] was conducted during the data analysis. The parameters were calculated according to the following equation:

(3) $$\langle v\rangle =\frac{\int_{f_{min}}^{f_{max}}f\cdot S\left(i_{ac2}\left(t\right)-i_{ac1}\left(t\right)\right)df}{\int_{f_{min}}^{f_{max}}S\left(i_{ac2}\left(t\right)-i_{ac1}\left(t\right)\right)df}.$$

Parameter 〈v〉 is described as the ratio of conventional perfusion parameter to concentration of moving red blood cells (CMBC) [43] estimated by the Doppler power spectrum, and is general expressed in arbitrary units.

**Figure 3:**
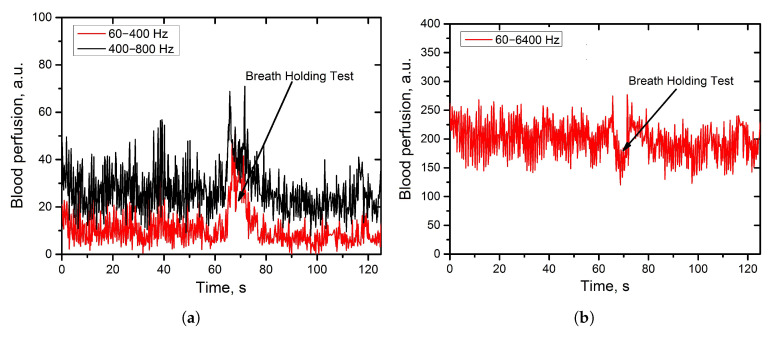
Registered skin blood perfusion during the breath-holding test evaluated by the integration in the sub-ranges of the photocurrent power spectrum. (**a**): blood perfusion from the low frequency range (60–400 Hz: red; 400–800 Hz-black); (**b**): blood perfusion from the broader frequency range (60–6400 Hz).

For the validation of the experimental setup, preliminary measurements have been done with the participation of healthy volunteers. The developed and implemented signal processing approach allowed us to reliably register and visualise (Figure 3) the effect of redistribution of the power spectrum signal due to the changes in the distribution of the RBCs speed in blood capillaries during a breath-holding test (BHT) [44]. In the middle of a deep breath, the level of the skin blood perfusion evaluated in the low-frequency range (60–400 Hz) increased prominently, while the overall blood perfusion figured out by the integration in the range of 60–6400 Hz decreased. The result is explained by the slowing down of the bulk of RBC during the procedure of the deep breath-holding causing the shift of the power spectrum to the region of the lower frequencies.

### 2.1. Research Protocol

**Figure 4:**
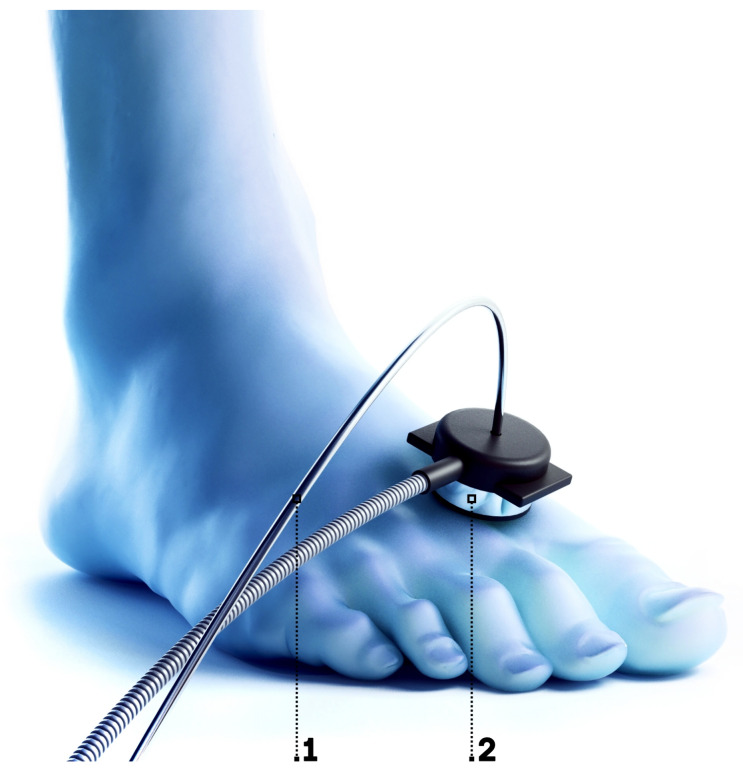
Allocation of the fibre optical probe combined with the attachment for the heat and cooling tests on the dorsal surface of foot. 1—fibre optical probe; 2—the attachment for the heating and cooling tests (contains Peltier element with water cooling).

The developed measuring setup has been validated in the frame of limited clinical studies with the involvement of patients with diabetic disorders. Thermal stimuli applied to the skin has been used to implement a study protocol, benefiting the most from the developed signal processing approach. The heat test is widely used in diagnostics of disorders associated with the regulation of blood perfusion. Moderate heating of the skin to the temperatures between 41–43 °C provokes activation of nociceptive C-fibres [45] and induces the release of nitric oxide [46] from the endothelial layer of vessels. The effect of the stimuli results in the increase of the skin blood perfusion and modulation of its oscillations. T2DM is often associated with an impairment in the response of blood perfusion on the thermal stimuli. The provocation test can also be applied together with a multi-modal approach that combines several measuring techniques such as laser Doppler flowmetry, diffuse reflectance measurements, or fluorescence spectroscopy [6]. Skin temperature dynamics during the skin heating has a significant diagnostic value, being supplemented with the blood perfusion measurements [47]. The fibre optic probe of the developed prototype of laser Doppler flowmeter with the bespoke signal processing algorithm was placed on the dorsal surface of the foot, and coaxially combined with attachment Figure 4 for the heating and cooling tests containing Peltier element with water cooling.

Reliable fixation on the probe on the foot was carried out using several mesh bandages. The measurements were taken 2 h after a meal and caffeine intake. The volunteers were acclimatised for at least 15 min to the conditions of the room where the measurements were conducted.

**Figure 5:**
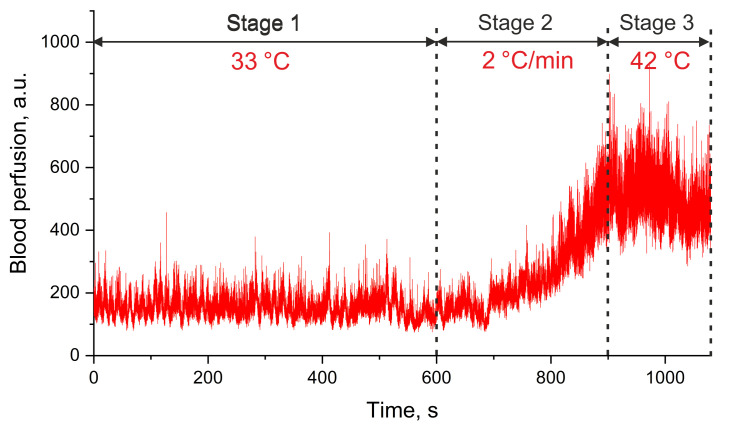
An exemplary trace of the blood perfusion during the implemented research protocol.

At the first stage (further on referred as stage 1), blood perfusion was recorded at a temperature of 33 °C for 10 min, to equalise the temperature conditions of the experiment for all subjects. At the second stage (in the text referred to as stage 2), the temperature was increased sequentially at a rate of 2 °C per min to 42 °C for 5 min. At the third stage (referred to below as stage 3), blood perfusion and effects occurring in the capillary blood flow due to heating were recorded for 3 min (Figure 5).

The cohort of conditionally healthy volunteers was divided into two groups consisting of 7 non-smoking volunteers aged 22 ± 0.5 years and (referred to as group 1) 6 volunteers (with 1 smoking person) aged 51 ± 6 (referred to as group 2), respectively. The group of patients comprised of 10 non-smoking volunteers aged 61 ± 7 with type 2 diabetes confirmed during for at least 5 years, but without diabetic foot, necrosis and visible lesions and characteristics described in Table 1.

All experiments were carried out at a temperature of 21–23 degrees, at a distance of 1 m from heat sources. Studies in patients and volunteers were conducted with the mandatory obtaining of informed consent for the participation in the study. The research protocol was approved by the Institutional ethical committee of Orel State University (Minutes No. 15 dated 21 February 2019).

### 2.2. Data Processing

**Figure 6:**
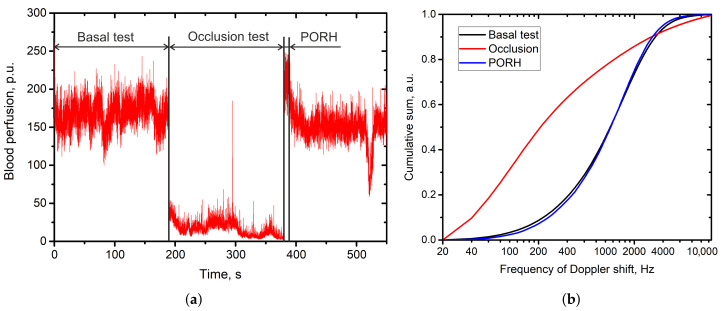
Cumulative sum curves calculated for the main stages of occlusion test. (**a**)—representative trace of the evaluated blood perfusion during the occlusion test; (**b**)—cumulative sum curves calculated for the stages of the occlusion test.

For the study, a novel approach was applied that previously was not used for signal processing in LDF. The implemented LDF channel (Figure 1) during the measuring procedure recorded the raw data with the power spectra from the photodiodes and performed blood perfusion calculation based on those measurements. To analyse the changes in a response to the applied functional tests, cumulative sum curves were calculated at the stage of post-processing by the following steps. Firstly, power spectra weighted by the frequency were calculated. Secondly, cumulative sum curves for every weighted power spectrum in samples were calculated according to the following recursive expression:

(4) $$C_{n}=C_{n-1}+\frac{f_{n}\cdot S\left(f_{n}\right)}{\sum f_{i}\cdot S\left(f_{i}\right)},$$

where $C_{n}$—a cumulative sum of the series from one to *n*; $C_{0}=0$; $f_{n}\cdot S\left(f_{n}\right)$—perfusion calculated for the frequency bin *n*; $\sum f_{i}\cdot S\left(f_{i}\right)$—perfusion calculated over all frequencies; *n* is changed in range from 1 to 640 that equals a frequency range from 0 to 12,800 Hz. Thirdly, the average cumulative sum curve is calculated for a specified period of the perfusion recording. $C_{n}$ is a value that represents what part of the signal is localised before the certain frequency. The shape of the cumulative sum curve depends on the distribution of the signal over the frequencies of Doppler broadening. The expression (3) can be expressed in an integral form that resembles Equation (1):

(5) $$C_{n}=\frac{\int_{f_{min}}^{f_{n}}f\cdot S\left(f\right)df}{\int_{f_{min}}^{f_{max}}f\cdot S\left(f\right)df},$$

where $f_{n}$ is the frequency of the *n*-th frequency bin. The processing of the data obtained in the standard occlusion test demonstrates the sensitivity of the calculated $C_{n}$ parameter to the changes in skin blood perfusion during the occlusion stage and stage of post-occlusive reactive hyperaemia (Figure 6).

The cumulative sum curve for the signal recording when occlusion is applied is growing faster. During the occlusion, there is a shift of the localisation of the signal to the low frequency band. Also the overall shape of the cumulative sum curve changes. To quantify the redistribution of the signal over the frequency subbands before and after provocative test, we calculate the parameter of the area enclosed between the two cumulative sum curves.

**Figure 7:**
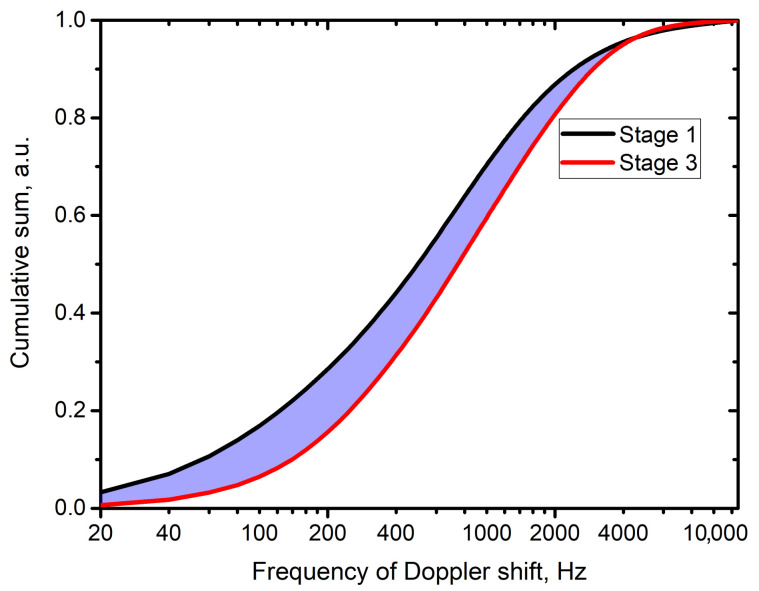
Representative example of cumulative sum curves and analysed area between them (highlighted in purple) for the stages 1 and 3 of the implemented research protocol with thermal stimuli applied to the skin

An example of the two curves calculated for stage 1 and stage 3 of the described protocol (Figure 5) implemented in patients is shown in Figure 7, where the purple highlighted area represents the introduced parameter, further called Area between Curves (AbC). The AbC parameter is calculated for the area between the curves from starting frequency to the first intersection of the curves. The area after the intersection is not included in the calculation.

It is known that the shape of a first moment of the power spectrum depends on the speed of moving red blood cells. However, it remains unclear which mechanisms are more responsible for perfusion alterations during functional tests—a proportional increase in amplitude or changes in the shape of weighted power spectra.

The second informative parameter used in the data analysis was the difference between averaged blood perfusion values calculated for stage 3 and stage 1 (subsequently called the DBP parameter):

(6) $$DBP={\langle PU\rangle }_{stage3}-{\langle PU\rangle }_{stage1},$$

where 〈...〉—symbol of averaging by time. The DBP parameter is often used in studies as a parameter characterising functional state of the microvascular regulation [20,48]. Also, the average RBC speed calculated according the expression (4) was estimated for the analysed stages 1 and 3:

(7) $${\langle v\rangle }_{31}={\langle v\rangle }_{3}-{\langle v\rangle }_{1},$$

where ${\langle v\rangle }_{1}$ and ${\langle v\rangle }_{3}$ — average estimation of blood cell speed calculated by whole duration of stage 1 and stage 3 correspondingly.

## 3. Results

**Figure 8:**
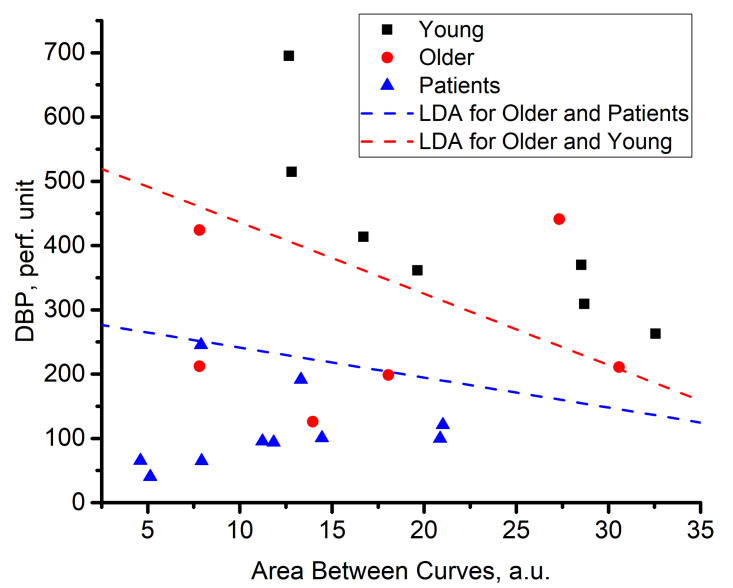
Scatter plots for analysed groups of patients and volunteers with the dividing lines of the LDA classifiers.

The approach of the cumulative sum curve analysis is intended to demonstrate that mean perfusion changes under thermal stimuli may have different characteristics from volunteer to volunteer in the context of power spectral distribution. Using the two parameters AbC and DBP as a feature space, two binary linear classifiers based on linear discriminant analysis (LDA) were implemented to distinguish the group of patients and the group of volunteers 2, (Y1) as well as the groups of volunteers 1 and 2 (Y2) (Figure 8).

The calculated discriminant functions have been identified to have the following form:

$$\begin{array}{c} Y1=-5.0622+0.84\cdot X1_{2p}+0.0177\cdot X2_{2p}\\ Y2=-4.28022+0.86\cdot X1_{12}+0.0076\cdot X2_{12} \end{array}$$

Table 1 shows the area under curve (AUC) values for the classifiers based on the discriminant functions calculated using only one of the two input parameters and for their combined use.

The scatter plots for the collected data in Figure 8 are divided into three areas. The first one, the lower-left corner of Figure 8 is represented by the group of patients. Group 1 and group 2 occupied the intermediate position and the upper-right corner, respectively. Group 2 and some individuals from the patient group are characterised by a high DPB parameter. Other individuals showed a relatively high value of the AbC parameter.

## 4. Discussion

The observed effects are potentially connected with the presence of two ways of mean blood perfusion increase. One of them occurs when the value of the AbC parameter is relatively high. Apparently, the increase in mean blood perfusion is connected with the redistribution of the spectrum amplitude to higher frequencies of Doppler broadening. Whereas, if the DBP parameter increases and the AbC parameter is small, then the blood perfusion changes due to a proportional amplification in the spectrum amplitudes, which will not drastically change the shape of the cumulative sum curves. Thus, the shape of the cumulative sum curve calculated during stage 3 is changed weakly. Consequently, the AbC parameter does not substantially increase. Group 1 is characterised by both mechanisms: a sufficient broadening of the spectrum, and a significant DBP parameter. In the case of group 2, the trend is not recognised. However, group 2 is characterised by reduced values of the DBP and significantly high scattering values relative to the AbC parameter. As for the patient group, both parameters are significantly reduced.

Variations in the AbC parameter can be associated with various factors. The article [49] presents the results of modelling the distribution of photons over the Doppler shift frequencies taking into account changes in both the speed of scattering particles and their concentration. The simulation results show that both of these parameters simultaneously influence the shape of the Doppler spectra. Indeed, during the thermal test, both an increase in the RBC concentration and a change in their average rate can occur. Moreover, article [30] described that the shape of power spectra depends on a distribution characteristic of RBC speed. It is difficult to say with complete certainty which of the described factors is decisive for a particular functional test in healthy volunteers.Further research and modelling is required since Figure 8 shows a substantial variation of the results from healthy volunteers. However, for patients with type 2 diabetes mellitus, it is important to note that changes in the shape of the power spectra in response to the heat test are weak in comparison with cohorts of healthy volunteers, which is demonstrated by the AbC.

Power spectrum shape-changing (consequently, changes in cumulative sum curves) can occur for several reasons. It was previously described that severe geometric shape changing in capillary loops happens in patients with T2DM [50]. On the example of nailfold capillaroscopy, giant capillaries, and an increase of the distance between capillaries, the appearance of convoluted and intersecting capillaries are shown. The article [51] described the speed of distribution in a healthy capillary loop. To the best of our knowledge, such a numerical experiment for pathological capillaries did not perform. However, in the presence of sharp turns, bends and intersections in a capillary loop, the blood flow will differ substantially from laminar flow. The diameter of capillaries and its influence on power spectra is also considered by Fredriksson et al. [52]. Possibly, the cross-section of capillary loops in patients with T2DM is larger than in healthy volunteers. Thus, a change in the geometric shape of the capillaries, the cross-section and a decrease in their number [53] lead to phenomena where an increase in blood perfusion occurs only due to a proportional increase in the power spectrum amplitudes with relatively constant overall shape of the function. From a clinical point of view, the low-values of parameters AbC and DBP in the cohort of patients can be observed due to the decreasing of capillary loop quantity and pathological changes in the capillaries where the RBC speed profile changes slightly during the applied thermal stimuli.

Thus, introduced in this study, AbC parameter is a novel approach for LDF signal processing. As seen for group 1 and 2 in Table 2, combined use of the parameters significantly increases the prediction value in comparison with the classifiers built using the parameters separately.

To date, there are several methods for recording speed-resolved blood perfusion. For example, PeriFlux 6000 EPOS (Perimed, Järfälla, Sweden) enable simultaneously registering blood perfusion in the frame of three speed ranges. This approach is based on a mathematical model that takes into account the concentration of RBC, blood oxygenation, the potential geometric shapes of capillaries, as well as the optical properties of biological tissues under study. These capabilities are achieved by combining the LDF with the diffuse reflectance spectroscopy measuring channel. A series of studies were made using that technique, for example by Wang et al. [54]. Nevertheless, the current study approach can be implemented using hardware of both classical LDF devices and ones implementing more advanced techniques with recording speed-resolved blood perfusion, supplementing the present arsenal of the signal processing methods in the field.

Calculated AUC-scores of 〈v〉-based classifiers and classifier with extended feature space for three involved parameters for group 2 vs. patients and group 1 vs. group 2 is shown in Table 2. The combined use of the three parameters augmented with parameter of blood perfusion improves the classification between the compared groups. The proposed estimates represent different aspects of blood perfusion and complement each other.

Thus, the introduced AbC parameter can be used for the interpretation of the origin of observing alterations in mean blood perfusion indicating whether the changes are due to the RBC speed redistribution, or due to the proportional increase in all components of blood perfusion. The AUC-score for the classifier between aged healthy volunteers and patients is of almost the same value as for using a single DBP parameter, which can also be explained by the limited size of the tested cohort of volunteers. Identification of factors, which affect the shape of perfusion distribution by Doppler frequencies, can be of particular interest in the diagnostics of disorders characterised by changes in blood perfusion. It is important to note that the cumulative sum-based estimations such as Area between Curves can be combined with conventional perfusion measurements (CMBC and blood cell speed 〈v〉). As is shown on Table 2, the combination of the proposed and conventional parameters is better suited for classification for both considered pairs.

The proposed approach can find applications in clinical practice and diagnostics without significant modifications of the standard configuration of LDF channel, facilitating the translation of the study results in routine diagnostics procedures.

## 5. Conclusions

In the study, the measurement of perfusion is complemented by a new parameter based on the calculation of cumulative sums from the power spectrum of the photodetector signals. The described diagnostic approach allows for revising the known protocols for characterising blood microcirculation parameters based on LDF measurements. In the context of the proposed signal processing technique, a new feature space can be added to the set of known diagnostic parameters expanding the range of applications for the LDF. The translation of the study results suggests that routine diagnostics procedures do not require significant modifications of the standard configuration of LDF channel. Also, the method does not involve any additional measurement channels (fluorescence, diffuse reflectance spectroscopy, and others) that still demonstrate relatively high accuracy of classification for the diagnostics of impaired blood microcirculation, even being tested in a limited size of the cohort of patients with T2DM.

## Figures and Tables

**Table 1:** Characteristics of T2DM patients and volunteers.

Parameters	Patients	Group 1	Group 2
Age (y)	61 ± 7 *	22 ± 0.5 *	51 ± 6 *
Sex (M/F)	3/7	2/5	3/3
Systolic BP (mmHg)	125 ± 11	122 ± 8	120 ± 5
Diastolic BP (mmHg)	75 ± 7	70 ± 3	77 ± 4
Body mass index (kg/m${}^{2}$)	31 ± 4.5 *	24 ± 3.5	25 ± 3.8
Fasting glucose (mmol/L)	10.4 ± 3	-	-
Diabetes duration (y)	11.5 ± 4	-	-
HbA1c (%)	7.1 ± 0.2	-	-
Total cholesterol (mmol/L)	5.2 ± 0.8	-	-
Creatinine (μmol/L)	78.6 ± 7.6	-	-
Urea (mmol/L)	6 ± 0.9	-	-
ALT (IU/L)	24.9 ± 5.4	-	-
AST (IU/L)	19.9 ± 4.3	-	-

Note: Data in the columns is represented as mean ± SD except Sex parameter. Reference values of the laboratory: HbA1c 4.0% to 6.0%, total cholesterol 3.5 to 5.0 mmol/L, urea 2.5 to 7.5 mmol/L, creatinine 70 to 110 μmol/L, ALT 10 to 38 IU/L, and AST 10 to 40 IU/L. *—denotes a statistical difference identified by Mann-Whitney test between two other groups, *p* < 0.05.

**Table 2:** AUC-scores for the tested classifiers for the groups of volunteers and patients.

Classifiers	AUC
Group 1 vs. Group 2	
DBP classifier	0.76
AbC classifier	0.64
${\langle v\rangle }_{31}$ classifier	0.79
Linear classifier (DBP and AbC together)	0.86
Linear classifier (DBP and ${\langle v\rangle }_{31}$ together)	0.91
Complex classifier (DBP, AbC, ${\langle v\rangle }_{31}$)	0.928
Group 2 vs. Patients	
DBP classifer	0.92
AbC classifer	0.65
${\langle v\rangle }_{31}$ classifier	0.71
Linear classifier (DBP and AbC together)	0.9
Linear classifier (DBP and ${\langle v\rangle }_{31}$ together)	0.91
Complex classifier (DBP, AbC, ${\langle v\rangle }_{31}$)	0.933
